# A versatile toolkit for overcoming AAV immunity

**DOI:** 10.3389/fimmu.2022.991832

**Published:** 2022-09-02

**Authors:** Xuefeng Li, Xiaoli Wei, Jinduan Lin, Li Ou

**Affiliations:** ^1^ The Sixth Affiliated Hospital of Guangzhou Medical University, Qingyuan People’s Hospital; State Key Laboratory of Respiratory Disease, Sino-French Hoffmann Institute, School of Basic Medical Sciences, Guangzhou Medical University, Guangzhou, China; ^2^ Shenzhen Luohu People’s Hospital, The Third Affiliated Hospital of Shenzhen University, Shenzhen, China; ^3^ Guangzhou Dezheng Biotechnology Co., Ltd., Guangzhou, China; ^4^ Genemagic Biosciences, Philadelphia, PA, United States; ^5^ Department of Pediatrics, University of Minnesota, Minneapolis, MN, United States

**Keywords:** adeno-associated virus (AAV), gene therapy, immunity, toxicity, immunosuppressant, capsid engineering, exosome, CpG

## Abstract

Recombinant adeno-associated virus (AAV) is a promising delivery vehicle for *in vivo* gene therapy and has been widely used in >200 clinical trials globally. There are already several approved gene therapy products, e.g., Luxturna and Zolgensma, highlighting the remarkable potential of AAV delivery. In the past, AAV has been seen as a relatively non-immunogenic vector associated with low risk of toxicity. However, an increasing number of recent studies indicate that immune responses against AAV and transgene products could be the bottleneck of AAV gene therapy. In clinical studies, pre-existing antibodies against AAV capsids exclude many patients from receiving the treatment as there is high prevalence of antibodies among humans. Moreover, immune response could lead to loss of efficacy over time and severe toxicity, manifested as liver enzyme elevations, kidney injury, and thrombocytopenia, resulting in deaths of non-human primates and patients. Therefore, extensive efforts have been attempted to address these issues, including capsid engineering, plasmapheresis, IgG proteases, CpG depletion, empty capsid decoy, exosome encapsulation, capsid variant switch, induction of regulatory T cells, and immunosuppressants. This review will discuss these methods in detail and highlight important milestones along the way.

## 1 Introduction

Adeno-associated virus (AAV) is a replication-defective virus that infects vertebrates, including human and non-human primates (1). The genome of AAV is approximately 4.8 kb of single-stranded DNA flanked by inverted terminal repeats (ITRs), which are essential for its life cycle (2). AAV has been associated with no known diseases for long, however, a recent study suggested a potential involvement of AAV in a hepatitis outbreak in Scotland (3). Due to its low pathogenicity and risk of insertional mutagenesis, AAV has been developed as ‘biological nanoparticles’ for *in vivo* delivery of gene therapy (4). Starting from the late 90s, there have been gene therapy clinical trials using AAV delivery (AAV gene therapy for cystic fibrosis) (5). Until now, there have been over 100 clinical trials using wild-type and engineered AAV vectors (6). The first approved AAV gene therapy drug was Glybera for treating lipoprotein lipase deficiency using AAV1 vector (7), which was approved in Europe in 2012. Then, Luxturna, an AAV2-based gene therapy for retinal diseases (8), was approved in the USA in 2017, and Zolgensma, an AAV9-based gene therapy for spinal muscular atrophy in the USA in 2019 (9). The clinical and commercial success of AAV gene therapy encouraged a rapid clinical development. However, among the ever-increasing clinical development of AAV gene therapy, serious adverse events and toxicity, mostly due to immunity elicited by AAV delivery, were observed. Safety concerns culminated when a clinical trial (NCT03199469) for X-linked myotubular myopathy (XLMTM) reported 4 patient deaths, presumably due to hepatotoxicity (10). Further, incidence of hepatocellular carcinoma (11), elevated liver enzymes (12), MRI abnormalities (13), and dorsal root ganglia (DRG) degeneration (14) were reported from multiple clinical trials. It is of particular interest to highlight patient death cases associated with AAV delivery (summarized in **Table 1**). Due to the small sample size, it may be premature to draw clear conclusions. Yet, these cases more frequently occurred in CNS and muscular diseases and were associated with a high dose. In light of this, FDA organized a two-day Cellular, Tissue, and Gene Therapies Advisory Committee (CTGAC) meeting focused on addressing AAV toxicity issues in 2021. In addition to toxicity, AAV immunity may be also responsible for loss of efficacy over time due to promoter silencing or T cell-mediated elimination of transgene-expressing cells (22).

**Table 1:** Patient death cases that occurred in AAV trials.

Disease	Drug name	Sponsor	AAV	Dose	ROA	Patient deaths	References
Inflammatory arthritis	tgAAC-94	Targeted Genetics Corporation	AAV2	1E11, 1E12, 1E13 vg/mL joint volume	Intraarticular	1	(15)
GAN	TSHA-120	NINDS	scAAV9	3.5E13, 1.2E14, 1.8E14, 3.5E14 vg	Intrathecal	1	(16)
DMD	PF-06939926	Pfizer	AAV9	1E14, 3E14 vg/kg	Intravenous	1	(17)
ALS	AAV-miR-SOD1	U Mass Medical School	AAVrh10	4.2E14 vg	Intrathecal	1	(14)
XLMTM	AT132	Astellas	AAV8	1E14, 3E14 vg/kg	Intravenous	4	(10)
SMA	Zolgensma	Novartis Gene Therapies	scAAV9	1.1E14 vg/kg	Intravenous	1 (2)*	(18)
MPS IIIA	SAF-302	Lysogene	AAVrh10	7.2E12 vg	Intracerebral	1	(19)
GM2 gangliosidosis	TSHA-101	Taysha	AAV9	Undisclosed	Intrathecal	1	(20)

*Two death cases occurred recently after Zolgensma is commercialized, but no detailed information was released yet (21). GAN, giant axonal neuropathy; DMD, Duchenne muscular dystrophy; ALS, amyotrophic lateral sclerosis; SMA, spinal muscular atrophy; MPS IIIA, mucopolysaccharidosis type IIIA.

## 2 Immune response against AAV

**Figure 1:**
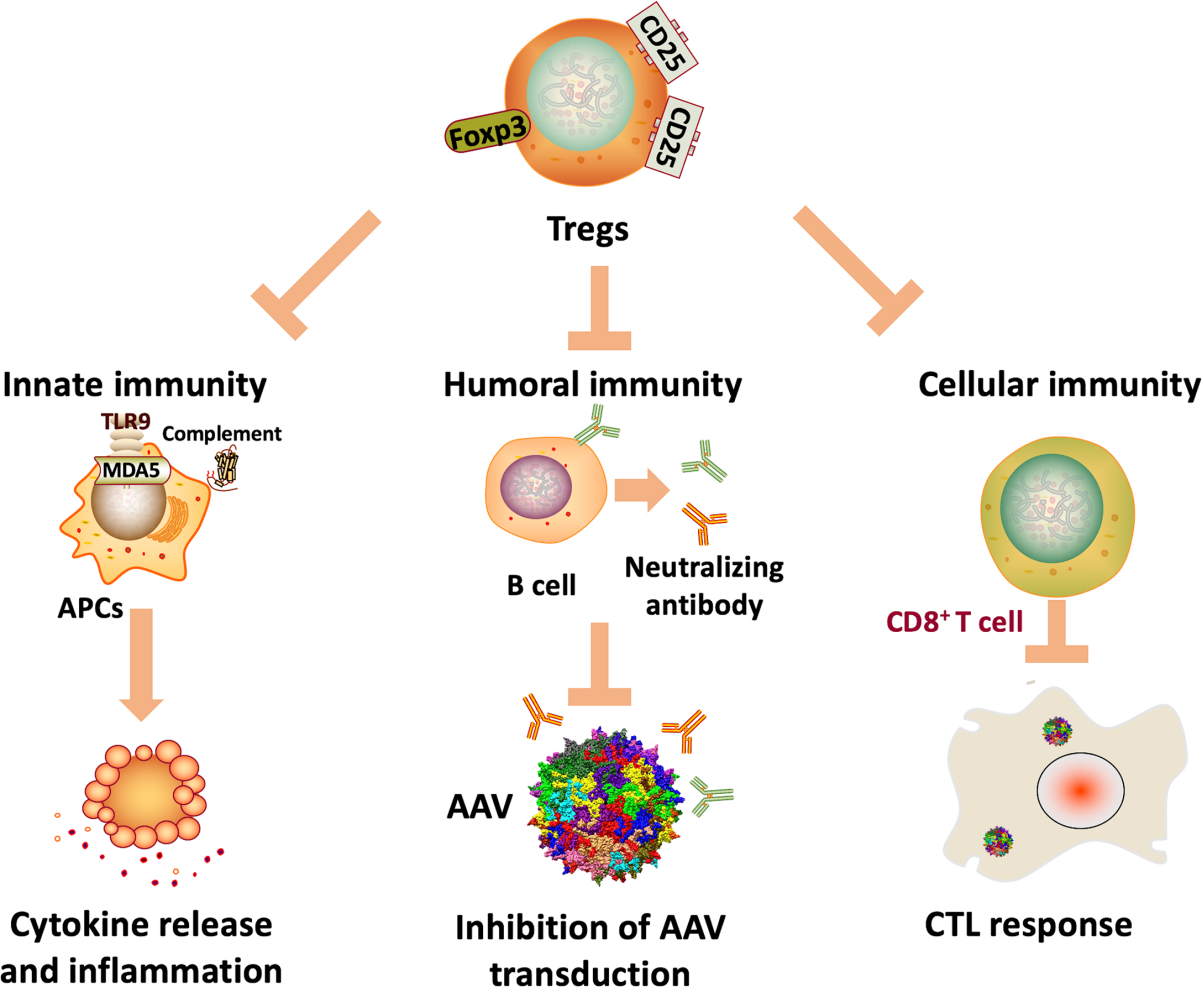
Potential mechanisms of AAV immunity. Innate immunity against AAV genome and transcripts can be activated by TLR9 and MDA5 recognition, resulting in cytokine release, inflammation, and ultimately CTL responses. In addition, the complement system may also be activated, which is associated with thrombotic microangiopathy and adverse events in clinical trials. Also, humoral immunity against capsids can significantly inhibit AAV transduction, limiting the application of AAV gene therapy in many patients. Further, cellular immunity against capsids and transgene products can eliminate transgene-expressing cells, ultimately resulting in loss of efficacy.

Immunity against AAV gene therapy is multidimensional, including innate, humoral, and cellular immune responses against capsids, genome sequences, and transgene products. Since the main focus of this review is on the toolkit to overcome AAV immunity, specific mechanisms of AAV immunity are only briefly discussed here as the context (illustrated in **Figure 1**). Action of innate immunity against AAV gene therapy was mediated through Toll-like receptor 9 (TLR9), which recognizes unmethylated CpG elements in AAV genome (23). The subsequent immunological cascades ultimately results in release of cytokines, inflammation and cytotoxic T cell (CTL) responses (24, 25). There is also evidence indicating the activation of the complement system, a key component of innate immunity, and renal injury in clinical studies (26, 27). Meanwhile, due to natural AAV infection, there is a high prevalence of pre-existing antibodies against AAV in humans (28). Pre-existing antibodies against capsids can significantly inhibit efficient AAV transduction in NHPs (29) and humans (12). Therefore, pre-existing antibodies prevent many patients from being eligible for potentially life-saving treatments. Moreover, humoral immunity against both capsids and transgene products elicited by AAV administration makes repeat dosing challenging (30). In addition, T cell responses may eliminate transgene-expressing cells, resulting in hepatotoxicity and loss of transgene expression in several clinical studies (12, 31–33). Fortunately, the immune response is not always against AAV-mediated gene transfer, evidenced by induction of regulatory T cells (Tregs) that suppress immune response and facilitate sustained transgene expression (34, 35).

## 3 Toolkit to overcome AAV immunity

There have been extensive efforts to address AAV immunity. In this review, a versatile toolkit for overcoming AAV immunity is discussed in an order of starting from methods that have more clinical applications. Within each section, preclinical data is discussed before clinical data, if any.

### 3.1 Immunosuppressants

#### 3.1.1 Corticosteroids

Corticosteroids (e.g., methylprednisolone, prednisolone, prednisone) are commonly used immunosuppressants for their global inhibitory effects on innate and adaptive immunity (36). Corticosteroids are most-widely used immunosuppressants for AAV gene therapy clinical studies (**Table 2**) and also used in approved gene therapy products, Luxturna (37) and Zolgensma (9). However, recent studies suggest that corticosteroids alone may not be sufficient. In the previously mentioned XLMTM trial (NCT03199469), there were 4 patient deaths, presumably resulting from hepatotoxicity (10). These patients receiving high dose IV administration AAV (1 to 3 x10^14^ vg/kg) were also on a 16-week prednisolone regimen. In another clinical study for ALS, one patient developed meningoradiculitis after intrathecal AAV delivery in spite of methylprednisolone and prednisone usage (14). In contrast, lower T cell responses and NAbs were observed in a second patient who was received a different immunosuppressant regimen that includes rituximab and sirolimus in addition to corticosteroids prior to AAV delivery, indicating the role of immunosuppressant selection and timing. The drawback of corticosteroids are side effects and non-specific immunosuppressing effects. In summary, due to the established safety profile and effects in preventing or alleviating immune responses, corticosteroids are the most frequently used immunosuppressants in AAV clinical studies.

**Table 2:** AAV clinical studies using immunosuppressants.

Disease	Immune suppression/modulation	Trial ID	Drug name	Serotype
Achromatopsia	Corticosteroids	NCT02599922	AGTC-401	AAV2
Achromatopsia	Corticosteroids	NCT02935517	AGTC-402	AAV2
Achromatopsia	Corticosteroids	NCT02610582	rAAV.hCNGA3	AAV8
Acute intermittent porphyria	Corticosteroids	NCT02082860	rAAV2/5-PBGD	AAV5
Alpha-1 antitrypsin deficiency	Corticosteroids	NCT02168686	ADVM-043	AAVrh10
Amyotrophic lateral sclerosis	Prednisone, methylprednisolone	N/A	AAV-miR-SOD1	AAVrh10
Becker muscular dystrophy	Corticosteroids	NCT01519349	AAV1-Follistatin	AAV1
Canavan	Prednisone	NCT04833907	rAAV-Olig001-ASPA	Undisclosed
Canavan	Rituximab, Sirolimus, Methylprednisolone	NCT05317780	rAAV9-CB6-AspA	AAV9
Choroideremia	Prednisolone	NCT01461213	BIIB111	AAV2
Choroideremia	Prednisolone	NCT02077361	BIIB111	AAV2
Choroideremia	Prednisolone/prednisone	NCT02407678	BIIB111	AAV2
Choroideremia	Prednisolone/prednisone, dexmethasone, Moxifloxacin	NCT02671539	BIIB111	AAV2
Danon	Sirolimus	NCT03882437	RP-A501	AAV9
Diabetic macular edema	Corticosteroids	NCT04418427	ADVM-022	AAV2.7m8
Duchenne muscular dystrophy	Corticosteroids	NCT00428935	d3990	AAV2.5
Duchenne muscular dystrophy	Corticosteroids	2020-002093-27	GNT0004	AAV8
Duchenne muscular dystrophy	Corticosteroids	NCT04281485	PF-06939926	AAV9
Duchenne muscular dystrophy	Corticosteroids	NCT02376816	SRP-9001	AAVrh74
Duchenne muscular dystrophy	Corticosteroids	NCT03333590	rAAVrh74.MCK. GALGT2	AAVrh74
Duchenne muscular dystrophy	Corticosteroids	NCT03375164	SRP-9001	AAVrh74
Duchenne muscular dystrophy	Corticosteroids	NCT03769116	SRP-9001	AAVrh74
Duchenne muscular dystrophy	Corticosteroids	NCT04626674	SRP-9001	AAVrh74
Duchenne muscular dystrophy	Corticosteroids	NCT05096221	SRP-9001	AAVrh74
Duchenne muscular dystrophy	Corticosteroids	NCT04240314	AT702	scAAV9
Duchenne muscular dystrophy	Corticosteroids, eculizumab	NCT03362502	PF-06939926	AAV9
Duchenne muscular dystrophy	Corticosteroids, eculizumab	NCT03368742	SGT-001	AAV9
Duchenne muscular dystrophy	Prednisone	NCT02354781	rAAV1.CMV. huFollistatin344	AAV1
Fabry	Corticosteroids	NCT04519749	4D-310	4D-A101
Fabry	Corticosteroids	NCT04046224	ST-920	AAV2/6
Fabry	Corticosteroids	NCT04040049	FLT190	AAV8
Frontaltemporal dementia	Corticosteroids	NCT04747431	PBFT02	AAVhu68
Frontaltemporal dementia	Methylprednisolone, Sirolimus, Prednisone, Rituximab	NCT04408625	PR006	AAV9
Giant axonal neuropathy	Corticosteroids	NCT02362438	TSHA-120	scAAV9
Glycogen storage disease	Corticosteroids	NCT03517085	DTX401	AAV8
GM1 gangliosidosis	Corticosteroids	NCT04713475	PBGM01	AAVhu68
GM1 gangliosidosis	Rituximab, Sirolimus, Methylprednisolone, Prednisone	NCT03952637	AXO-AAV-GM1	AAV9
GM2 gangliosidosis	Corticosteroids	NCT04669535	AXO-AAV-GM2	AAVrh8
Hemophilia A	Corticosteroids	NCT03003533	SPK-8011	AAV-LK03
Hemophilia A	Corticosteroids	NCT03061201	SB-525	AAV2/6
Hemophilia A	Corticosteroids	NCT03001830	GO-8	AAV2/8
Hemophilia A	Corticosteroids	NCT02576795	Roctavian	AAV5
Hemophilia A	Corticosteroids	NCT03370913	Roctavian	AAV5
Hemophilia A	Corticosteroids	NCT03520712	BMN 270	AAV5
Hemophilia A	Corticosteroids	NCT03392974	Roctavian	AAV5
Hemophilia A	Prednisolone	NCT03588299	DTX201	AAVhu37
Hemophilia A	Prednisone, prednisolone	NCT03370172	BAX 888	AAV8
Hemophilia B	Corticosteroids	NCT02484092	SPK-9001	AAV-Spark100
Hemophilia B	Corticosteroids	NCT03307980	SPK-9001	AAV-Spark100
Hemophilia B	Corticosteroids	NCT02695160	SB-FIX	AAV2/6
Hemophilia B	Corticosteroids	NCT03569891	AMT-061	AAV5
Hemophilia B	Corticosteroids	NCT02396342	AMT-060	AAV5
Hemophilia B	Corticosteroids	NCT03489291	AMT-061	AAV5
Hemophilia B	Corticosteroids	NCT01687608	AskBio009 (BAX 335)	AAV8
Hemophilia B	Corticosteroids	NCT02618915	DTX101	AAVrh10
Hemophilia B	Corticosteroids	NCT00979238	scAAV2/8-LP1- hFIXco	scAAV2/8
Hemophilia B	Corticosteroids, tacrolimus	NCT03369444	FLT180a	AAVS3
Hemophilia B	Prednisolone	NCT01620801	SPK-9001	AAV8
Leber congenital amaurosis	Corticosteroids	NCT00643747	tgAAG76	AAV2
Leber congenital amaurosis	Corticosteroids	NCT00516477	Luxturna	AAV2
Leber congenital amaurosis	Corticosteroids	NCT00481546	rAAV2-CBSB-hRPE65	AAV2
Leber congenital amaurosis	Corticosteroids	NCT00999609	Luxturna	AAV2
Leber congenital amaurosis	Corticosteroids	NCT01208389	Luxturna	AAV2
Leber congenital amaurosis	Prednisone, Triamcinolone acetonide	NCT03920007	SAR439483	AAV5
Limb-girdle muscular dystrophy	Corticosteroids	NCT05230459	LION-101	AAV9
Limb-girdle muscular dystrophy	Prednisone	NCT03652259	SRP-9003	AAVrh74
Leber hereditary optic neuropathy	Corticosteroids	NCT02652767	GS010	AAV2
Leber hereditary optic neuropathy	Corticosteroids	NCT02161380	scAAV2-P1ND4v2	scAAV2tYF
Leber hereditary optic neuropathy	Corticosteroids, anti-inflammatory agent	NCT02064569	GS010	AAV2
Lipoprotein lipase deficiency	Mycophenolate mofetil, cyclosporine	NCT01109498	Glybera	AAV1
Lipoprotein lipase deficiency	Mycophenolate mofetil, cyclosporine, methylprednisolone	NCT00891306	Glybera	AAV1
Lipoprotein lipase deficiency	Mycophenolate mofetil, cyclosporine, prednisolone	NCT02904772	Glybera	AAV1
Metachromatic leukodystrophy	Corticosteroids	NCT01801709	AAVrh.10cuARSA	AAVrh10
Methylmalonic acidemia	Corticosteroids	NCT04581785	LB-001	LK03
Mucopolysaccharidosis type I	Corticosteroids	NCT02702115	SB-318	AAV2/6
Mucopolysaccharidosis type II	Corticosteroids	NCT03566043	RGX-121	AAV9
Mucopolysaccharidosis type II	Methyprednisolone, prednisone, sirolimus, tacrolimus	NCT05238324	HMI-203	AAVHSC
Mucopolysaccharidosis type II	Prednisone	NCT03041324	SB-913	AAV2/6
Mucopolysaccharidosis type IIIA	Tacrolimus, mycophenolate mofetil, steroids	NCT03612869	SAF-302	AAVrh10
Mucopolysaccharidosis type IIIB	Tacrolimus, mycophenolate mofetil	NCT03300453	AMT110	AAV2/5
Mucopolysaccharidosis type VI	Corticosteroids	NCT03173521	AAV2/8.TBG. hARSB	AAV2/8
Neovascular age-related macular degeneration	Corticosteroids	NCT01024998	AAV2-sFLT01	AAV2
Neovascular age-related macular degeneration	Corticosteroids	NCT03748784	ADVM-022	AAV2.7m8
Neovascular age-related macular degeneration	Prednisolone	NCT03585556	HMR59	AAV2
Ornithine transcarbamylase deficiency	Prednisone	NCT02991144	DTX301	scAAV8
Parkinson’s disease	Methylprednisolone, Sirolimus, Prednisone, Rituximab	NCT04127578	PR001	AAV9
Phenylketonuria	Corticosteroids	NCT03952156	HMI-102	AAVHSC15
Pompe	Corticosteroids	NCT04174105	AT845	AAV8
Pompe	Prednisone	NCT03533673	ACTUS-101	AAV2/8
Pompe	Rituximab, Sirolimus	NCT02240407	rAAV9-DES-hGAA	AAV9
Pompe	Rituximab, Sirolimus, Corticosteroids	NCT00976352	rAAV1-CMV-GAA	AAV1
Spinal muscular atrophy	Corticosteroids, eculizumab	NCT03381729	Zolgensma	scAAV9
Spinal muscular atrophy	Corticosteroids, eculizumab	NCT02122952	Zolgensma	scAAV9
Spinal muscular atrophy	Corticosteroids, eculizumab	NCT03306277	Zolgensma	scAAV9
Spinal muscular atrophy	Corticosteroids, eculizumab	NCT03461289	Zolgensma	scAAV9
Spinal muscular atrophy	Corticosteroids, eculizumab	NCT03505099	Zolgensma	scAAV9
Spinal muscular atrophy	Corticosteroids, eculizumab	NCT03837184	Zolgensma	scAAV9
Spinal muscular atrophy	Corticosteroids, eculizumab	NCT05073133	AVXS-101	scAAV9
X-linked myotubular myopathy	Prednisolone	NCT03199469	AT132	AAV8
X-linked retinoschisis	Corticosteroids	NCT02317887	AAV8-scRS/IRBPhRS	AAV8
X-linked retinitis pigmentosa	Corticosteroids	NCT03116113	BIIB112	AAV8

#### 3.1.2 Rapamycin

Rapamycin/sirolimus is a macrolide immunosuppressant that inhibits T and B cell activation and induce Tregs through targeting mTOR (38). As shown in one study (39), intraperitoneal administration of rapamycin and prednisolone reduced serum AAV9 NAbs by up to 93% and inhibited activation of B and T cells. Rapamycin/sirolimus was also frequently used in AAV clinical trials for immune suppression (e.g., NCT03882437, NCT02240407). Further, tolerogenic rapamycin nanoparticles, but not rapamycin in free form, have been shown to be able to inhibit both cellular and humoral immune response and induce Tregs in mice (40). A more recent study showed that tolerogenic rapamycin nanoparticles (SVP-rapamycin) prevented anti-capsid humoral and cellular responses, and more importantly, enabled AAV readministration in mice and NHPs (41). SVP-rapamycin also inhibited expansion of antigen-specific B cells. Notably, this effect was antigen-specific as coadministration of SVP-rapamycin (later known as ImmTOR) and AAV8 did not inhibit immunity against AAV5, which is preferable over other non-specific methods (e.g., IgG protease, plasmapheresis. ImmTOR has also been tested in clinical trials. A Phase Ib trial (NCT02648269) is testing whether ImmTOR could induce tolerance of pegadricase, a highly immunogenic enzyme derived from fungus, in hyperuricemia patients. In this study, ImmTOR has been shown to be able to inhibit anti-drug antibody formation in a dose-dependent manner (42). ImmTOR also showed a good safety profile as no treatment emergent serious adverse events (TESAEs) were observed.

#### 3.1.3 Mycophenolate mofetil

Another commonly used immunosuppressant for AAV clinical studies is mycophenolate mofetil (MMF), a compound that inhibits type II inosine monophosphate dehydrogenase and thus suppresses proliferation of T and B cells (43). It was shown that although MMF reduced anti-AAV1 humoral immunity, it also inhibited transduction of ssAAV1 (but not scAAV1) by impairing second strand DNA synthesis in rats (44). This highlights the need to consider using MMF as immunosuppressants for clinical trials using ssAAV delivery. Notably, MMF also induced mild leukopenia and body weight loss. Interestingly, when tested in combination with tacrolimus in NHPs, MMF did not inhibit liver transduction of AAV8 (45). Further studies are needed to understand whether this transduction-inhibitory effects of MMF is species- or serotype-specific.

#### 3.1.4 Complement inhibitors

Complement activation was recently found to be associated with adverse events in AAV clinical trials for DMD (26, 27) and SMA (46). Thus, there has been growing interest in applying complement inhibitors as immunosuppressants. Several FDA-approved complement pathway inhibitors could be potentially applied to inhibit AAV immunity and minimize risk of adverse events. For instance, C1 inhibitor effectively blocked AAV-induced complement activation in both human and mouse serum and whole blood samples. However, the complement blockade did not result in hampering the pro-inflammatory cytokine response towards AAV (47). Also, it had limited effect on anti-AAV antibodies. C5 inhibitor, eculizumab, has also been used in clinical trials to alleviate symptoms of complement activation, resulting in resolved toxicity in most patients (SGT-001, PF-06939926). In addition, Apellis Pharmaceuticals is applying C3 inhibitor, APL-9, to control the complement system during AAV delivery (48).

#### 3.1.5 mAbs

Rituximab is an anti-CD20 mAb that depletes B cells by inducing apoptosis (49). The combined use of rituximab and T helper cell inhibitor ciclosporin depleted antibody against transgene products and B cells in NHPs that had previously received AAV8 administration (50). This immunosuppressant combination also enabled efficient transgene expression after AAV readministration. Rituximab was also used in combination with methotrexate (a cytotoxic drug with antiproliferative effects on both B and T cells) in human rheumatoid arthritis patients (51). However, NAb titer drop was only observed in patients with a titer of <1:1,000. Moreover, the reduction was partial because the NAb titer in most of those patients remained to be above 1:5. Other mAbs have been assessed for suppressing AAV immunity. In one study (52), the combined use of rituximab and rapamycin successfully induced immune tolerance of transgene expression from AAV delivery in hemophilia A mice. Also, only mice receiving both rituximab and rapamycin had no increased inhibitors following rechallenge with intravenous FVIII protein therapy. This strategy is being tested in a clinical trial for Pompe disease (NCT02240407) (53). Preliminary results from this trial showed that the combined use of rituximab and rapamycin prevented humoral immune response and enabled AAV readministration (54). The combination of rituximab, sirolimus, and corticosteroids was also used in a recent clinical trial for GM2-gangliosidosis through CNS-directed AAV delivery (55). No vector-related adverse events was observed. In addition, since CD40/CD40L has been shown be involved in activation of CTL response by AAV (56), blocking this co-stimulatory pathway can be utilized for suppressing immune response against AAV delivery. For instance, dapirolizumab pegol, antibody against CD40 that is being tested in a Phase III trial for systemic lupus erythematosus (NCT04294667), can be used for this purpose. Similarly, CTLA4-IgG (abatacept) can block CD28-mediated immune activation by AAV and thus reduce capsid-specific CTL responses (57). In this study, CTLA4-IgG also inhibited formation of NAbs and enabled a second administration of AAV8 in a murine model of hemophilia B.

#### 3.1.6 Calcineurin inhibitors

Calcineurin inhibitors, including ciclosporin and tacrolimus, inhibit T cell proliferation and activation through targeting IL-2 (58). In one NHP study using intramuscular delivery of AAV8 or AAV9, tacrolimus significantly prolonged transgene expression from less than 8 weeks to beyond 42 weeks (59). However, one study showed that ciclosporin and tacrolimus inhibited Tregs proliferation and activation *in vitro* (60). Similarly, another study in human patients found that tacrolimus impaired Tregs proliferation, function, and phenotype, while rapamycin had beneficial effects on Tregs (61). In contrast, the combined use of tacrolimus and anti-CD4 antibody reduced antibodies against AAV capsids and transgene products and induced immune tolerance in mice (62). However, these results could not completely rule out the possibility of the inhibitory effects of tacrolimus on Tregs because tacrolimus was used in combination with another immunosuppressant in this study. Ciclosporin and MMF were used in combination in clinical studies of Glybera, which was the first approved gene therapy product in Europe (7, 63). In all patients, anti-AAV antibodies were not affected by immunosuppressants, and T cell response was observed in most patients (7). When the immunosuppression protocol changed the starting date and added methylprednisolone to the combination (63), humoral and cellular responses remained the same as the previous study. Therefore, in light of mixed results, the usage of calcineurin inhibitors as immunosuppressants for AAV gene therapy warrants further studies.

#### 3.1.7 Other pharmacological agents in preclinical development

Ubiquitination of AAV leads to proteasome-mediated degradation, antigen presentation of capsid-derived peptides, and immune activation (64). Thus, there have been efforts to use proteasome inhibitors to reduce AAV immunogenicity. One study showed that an FDA-approved proteasome inhibitor bortezomib reduced antigen presentation *in vitro* and enhanced transgene expression from AAV2, but not AAV8, in mice (65). Two other studies using AAV8 showed that bortezomib improved transduction in mice (66) and dogs (67). Another proteasome inhibitor, carfilzomib, also enhanced AAV2 transduction in mice, however, to a lesser extent than bortezomib (68). Nevertheless, bortezomib failed to enhance AAV9 transduction in rats (69). The mixed results of bortezomib may result from differences in serotypes, species, AAV dose, and bortezomib dose. In addition, although there were no toxicity findings in these preclinical studies, bortezomib use in humans was associated with peripheral neuropathy, thrombocytopenia, and neutropenia (70). Therefore, further studies are needed for clinical translation of this approach. Agents associated with oxidizing and anti-oxidizing pathways can be also applied as immunosuppressants for AAV gene therapy. Arsenic trioxide (As (2)O (3)), one oxidizing agent and FDA-approved chemotherapeutic drug, has been shown to stabilize AAV by reducing proteasome-mediated degradation (71). As mentioned earlier, AAV degradation resulted in increased antigen presentation and immune activation. Intraperitoneal administration of arsenic trioxide enhanced transduction and transgene expression after intravenous AAV delivery in mice (71). In addition, a synthetic antioxidant MnTBAP inhibited CD4^+^ T cell mediated immunity in mice after intramuscular AAV delivery in mice (72). MnTBAP functioned through downregulating and inducing reverse internalization of CD4. MnTBAP treatment prevented anti-AAV and anti-transgene cellular immunity, enabling readministration of the same serotype.

Cyclophosphamide, a potent immunosuppressant that inhibits both T and B cell immunity, was used in preclinical AAV studies (73, 74). In a gene therapy study for mucopolysaccharidosis type I (MPS I), after IV AAV6 delivery, transgene expression from a subset of mice reduced to 0 between 3 and 6 weeks post injection (74). When biweekly administration of cyclophosphamide was performed, sustained transgene expression was observed till 120 days. The effects of cyclophosphamide may be towards immunity against transgene products, not capsids, because a parallel study in MPS II mice using the same approach did not observe transgene expression reduction (75). A more recent study using a similar approach but CpG-removed transgene observed no reduction in transgene expression in MPS I mice (76), providing further supporting evidence.

Hydroxychloroquine (HCQ) is an approved anti-malaria drug, and accumulation of hydroxychloroquine inhibits the binding ability of TLR9 to DNA and subsequent immune activation (77). Subretinal AAV administration elicited innate immunity, evidenced by increased expression of IFN-γ, TNF-α, and CXCL10 (78). In this study, HCQ enhanced AAV transduction in NHP retinal pigment epithelial cells and human retina *ex vivo*. Further, coadministration of AAV with HCQ enhanced mouse retina transduction *in vivo*, and no evidence of toxicity was observed. It is noteworthy that high-dose systemic administration of HCQ was associated with risk of retinopathies.

#### 3.1.8 Key considerations for immunosuppressant usage

Overall, the timing, duration, and selection of immunosuppressants should be carefully considered. One NHP study showed that the addition of anti-IL-2 receptor antibody (daclizumab) to MMF and sirolimus failed to reduce antibody formation after AAV delivery (31). In contrast, MMF and sirolimus alone successfully reduced antibody formation, which may be explained by the fact that daclizumab had negative impacts on Tregs. A 5-drug immunosuppression regimen, including anti-thymocyte globulin (ATG), tacrolimus, rituximab, MMF, and methylprednisolone, inhibited T cell response after IV AAV5 delivery in NHPs (79). Interestingly, NAb formation occurred upon the removal of immunosuppression. Another NHP study showed that only delayed administration of ATG inhibited anti-drug antibody formation after AAV delivery, while early administration did not (80). FDA recently put a clinical trial for phenylketonuria on hold and lifted the hold after the immunosuppressant protocol incorporates tacrolimus and a shorter course of steroids (81). Other important things when considering immunosuppression regimens include side effects (e.g., infection) and the lack of suitable animal models for investigating and predicting AAV immunity due to the species difference.

### 3.2 Capsid engineering

#### 3.2.1 Rational design

Capsid engineering for capsids with resistance to antibodies can be performed through rational design (82–88). As shown in previous studies, based on understanding of the epitope responsible for antibody binding, mutations and insertions were introduced to disrupt antibody binding and thus generate novel capsids with reduced antibody affinity (82, 87–89). One caveat is that epitopes on capsids may be adjacent to regions that are important for other functions, e.g., packaging, intracellular transport. Therefore, impacts on normal functions and overall performance of AAV should be assessed during the process of discovering immune-escaping capsids through this approach. A novel capsid CAM130 had reduced NAb affinity against AAV1, but maintained comparable titer, transduction efficiency, and tissue tropism (88). A chimeric capsid, AAV2.5, was generated by inserting 5 amino acids of AAV1 into AAV2 (83). AAV2.5 maintained muscle tropism of AAV1 and receptor binding ability of AAV2 and also had reduced antigen presentation against both AAV1 and AAV2. In this study, AAV2.5 also enabled repeated administration in mice. Further, some wildtype serotypes have lower prevalence of NAbs in humans (28), and thus can be used as the template for capsid engineering. One example is mutagenesis of epitope regions of AAVrh10, which is one of the least seropositive common variants (82). The novel capsid AAVrh10-S671A achieved higher transduction efficiency and was at least 27-fold more resistant to NAbs when tested in mice. In addition, immune-escaping capsids were identified through incubating the rationale-designed AAV library with AAV2 neutralizing antibodies in serum or human immunoglobin (IVIG) (90). The packaging ability, infectivity, and tropism of top candidates were assessed as well. The drawback of this rational design-based approach is its dependence on structural understanding of AAV and identification of antibody-binding epitopes.

In addition, rational design can be also used in capsid engineering for reduced antigen presentations and cellular immunity. After entry into cells, AAV cells can be ubiquitinated for degradation in proteasomes (91), generating increased amount of antigens for immune activation. Since surface-exposed residues on AAV capsids can be ubiquitinated through phosphorylation, mutations of these residues (e.g. tyrosine, lysine, serine) to reduce the likelihood of ubiquitination can generate novel capsids with reduced ubiquitination, antigen presentation, and activated cellular immunity (92–99). One study identified AAV2(Y-F), a novel capsid generated through phenylalanine (F) for tyrosine (Y) substitutions of several surface-expose tyrosine residues, which achieved more sustained transgene expression and reduced CTL responses against transduced hepatocytes than AAV8 in mice (92). A triple Y-to-F mutant of AAV2, termed as AAV2tYF (99), has been applied in four clinical trials for ocular indications (NCT02416622, NCT02599922, NCT03316560, NCT02161380). In addition, mutations of lysine residues of AAV8 led to the identification of AAV-K137R, which had reduced antigen presentation, activation of innate immunity, and NAb formation (97). AAV-K137R also exhibited improved liver transduction in mice.

#### 3.2.2 Directed evolution

Another major category for capsid engineering is directed evolution, a method that mimics the process of natural selection to optimize certain features of DNA or proteins. Methods of AAV directed evolution include error-prone PCR (100–103), random peptide display (104–106), and DNA shuffling (107–110). Liver toxicity has been a major challenge for systemic AAV delivery, evidenced by incidence of liver enzyme elevations. There have been efforts in identifying liver-detargeting capsids for indications that liver is not the primary tissue target (111–113). Through random peptide display, a novel capsid B10 was identified (114). B10 had the superior brain tropism than AAV9 and had significantly reduced liver biodistribution after intravenous administration in mice and marmosets. Similarly, AAVMYO, a novel capsid with improved muscle tropism, was generated through random peptide display (115). AAVMYO was also 9-fold detargeted from liver than AAV9 after intravenous administration in mice. Another way of directed evolution is DNA shuffling, which generates a chimeric capsid containing sequence of multiple wildtype capsid variants. A novel capsid AAV-DJ was generated through DNA shuffling and subsequent high throughput screening in mice (106). AAV-DJ was able to transduce hepatocytes, more efficiently than parental serotypes, in the presence of intravenous human IVIG. A more recent study using the same approach identified a muscle-tropic capsid with improved resistance to NAbs, and the threefold symmetry region of AAV was deemed to be responsible for NAb recognition (116). Another chimeric capsid, SCH9, efficiently transduced neural stem cells and Purkinje cells and mediated higher transgene expression than AAV9 when delivered to mouse brains (117). Moreover, SCH9 showed enhanced resistance to NAbs and comparable packaging efficiency to wildtype capsid variants. In addition, one study screened novel liver-tropic capsids generated by DNA shuffling in a humanized mouse model (118). The library was incubated with human IgG to apply selective pressure for immune-evasive capsids. The top candidate AAV-NP59 was highly specific for human hepatocytes in this xenograft model. AAV-NP59 also elicited reduced anti-AAV IgG than AAV-LK03 (119) and AAV8, but not as good as previously discovered engineer capsid AAV-DJ.

### 3.3 Methods to overcome capsids-specific antibodies

#### 3.3.1 IgG proteases

It has been previously reported that even low levels of neutralizing antibodies against AAV (1:5–1:10) can completely abrogate transduction with high-titer vectors (12, 46, 120). Also, a substantial subset of humans have been exposed to AAV and have pre-existing NAbs (28). Therefore, NAbs significantly limits the potential of AAV gene therapy due to the exclusion of many patients from treatment. To this end, IgG proteases have attracted attention from gene therapists over the few years (121, 122). Prophylactic administration of IdeZ, an immunoglobulin-degrading enzyme from *Streptococcus equi subspecies zooepidemicus*, degraded IgG, prevented antibody neutralization, and rescued AAV transduction in passively immunized mice and NHPs with pre-existing NAbs (122). Similarly, IdeS is an endopeptidase able to degrade circulating IgG that is currently being tested in transplant patients (123). IdeS treatment allowed for efficient transduction in NHPs in the presence of pre-existing NAbs, and more importantly, AAV readministration (121). However, IdeS and IdeZ originate from bacteria, and thus it is likely that patients have pre-existing antibodies against IdeS and IdeZ, which may affect the cleavage efficiency. Therefore, one drawback of this approach is that administration of IgG proteases can be performed only once because of antibody formation against these enzymes. There have been efforts to develop methods that enable redosing of IgG proteases through ImmTOR (124). However, these IgG degrading enzymes can only address NAbs, but leave memory T and B cells intact, which can lead to low transduction, reduced durability, inflammation, and adverse events.

#### 3.3.2 Plasmapheresis

One way to reduce the inhibitory effects of pre-existing NAbs is plasmapheresis, a method to remove certain substances from the blood. Plasmapheresis is a widely-used and generally safe procedure in adults and children, even in pathological conditions. In one NHP study, plasmapheresis in sero-positive animals achieved efficient transduction, comparable to sero-negative animals and significantly higher than sero-positive animals without plasmapheresis (125). Also, there was no histopathological evidence of necrosis or lymphocyte infiltration in animals receiving plasmapheresis and AAVrh74 delivery. Plasmapheresis has also been tested in a clinical study, in which 3-5 consecutive plasmapheresis significantly reduced NAb titers in patients (126). The main drawback of plasmapheresis is non-specific removal of all circulating IgG, resulting in hypogammaglobulinemia and thus risk of infection. To this end, previous studies developed a capsid-specific plasmapheresis method using immunoadsorption (127–129). AAV-specific plasmapheresis column was used, enabling near-complete specific removal of anti-AAV IgG and AAV readministration in mice (127), rats (128), and NHPs (129). In addition, when plasmapheresis depletes antibodies in plasma, a quick rebound may come from antibodies in interstitial fluid and *de novo* production of B cells and antibodies (128). Therefore, AAV delivery should be performed in a short period post plasmapheresis. Another drawback is the need of repeated cycles to deplete IgG.

#### 3.3.3 Empty capsid decoy

Empty capsids are AAV vectors without DNA enclosed and usually side products during AAV packaging. Interestingly, empty capsids were used as decoy for anti-AAV antibodies, and thus allowed for efficient transduction of genome-containing capsids and transgene expression in mice and NHPs (130). Remarkably, even in the presence of high NAb titer (1:3,000), addition of empty capsids achieved ~45% of transgene expression from the control group that did not receive IVIG. Since empty capsids had no or minimal DNA inside, they are not expected to activate TLR9-mediated innate immunity. One concern about this approach is the increase in antigen load and subsequent T cell activation. To this end, mutation was introduced into the receptor binding site of capsid to generate an empty capsid mutant, which could not enter target cells but maintained the ability to absorb antibodies (130). By this means, antigen presentation by transduced cells would be reduced. However, increase in antigen presentation still seems to be inevitable because empty mutant capsid can be uptaken by APCs through receptor-independent pathways, e.g., pinocytosis. One study showed efficient antigen presentation from empty capsids (131). Similar findings were also observed in a murine model of rheumatoid arthritis (132), which showed the addition of empty capsids improved transduction and transgene expression. Another study showed that partially empty capsids containing fragmented genome sequence cogenerated with capsids containing full length genome sequence reduced liver transduction of AAV8 and also caused liver enzyme elevation in mice because they still contain DNA sequences (133). Therefore, it was recommended that decoy capsids should be packaged separately to generate completely empty capsids (133). It was also argued that for certain diseases, e.g., retina, where NAbs and synovial macrophages are absent, empty capsids should be avoided; but for cases like arthritic joints, empty decoys may be considered (134). However, this approach is not widely adopted in clinical studies. This is probably due to the requirement of large quantities of empty capsids (10-fold of full capsids), which creates additional manufacturing and regulatory challenges, and the increased antigen presentation in spite of mutations introduced to inhibit cell entry. Under most circumstances, the presence of empty capsids in clinical grade vectors is undesirable.

#### 3.3.4 Chemical modification of capsids

Another strategy to overcome NAbs is to encapsulate AAV capsids with polymers. One of the first efforts was not particularly successful. One study using polyethylene glycol (PEG) showed a modest improvement in resistance against NAbs at the price of significantly reduced infectivity (135). Another study using glycation on arginine and lysine residues showed that although binding affinity to antibodies decreased by 2 fold, transduction efficiency was reduced by 1,000 fold *in vitro*, potentially due to the disruption of AAV binding to heparin sulfate (136). Interestingly, as shown in one study, chemical modification led to redirection of tropism from liver to muscle. PEG conjugation activated by tresyl chloride (TMPEG) maintained tissue tropism to liver and muscle and achieved enhanced resistance to NAbs (137). TMPEG conjugation also enabled repeated AAV administration in mice. N-acetylgalactosamine (GalNAc), a ligand targeting hepatocytes, was also conjugated to lysine residues of AAV capsids (138). Significant lower total antibodies and NAbs were observed in mice receiving GalNAc-AAV. A more recent study applied click chemistry to specifically introduce unnatural amino acids (UAA), which can be tethered with various small molecules and polymers, into capsid proteins (139). This study showed that the resultant chemically modified AAV, oligo-AAV, had improved resistance against NAbs compared with previous polymer-conjugated AAVs. However, the packaging yields were significantly lowered. Similarly, another study using the PEGylation at UAA site strategy encountered the issue of decreased titer as well, although antibody recognition was modestly improved (140).

#### 3.3.5 Exo-AAV

Exosome, natural extracellular vesicles secreted from cells, is being developed as non-viral delivery vectors of proteins or nucleic acids (141). As shown in one study (142), exosome-associated AAV (exo-AAV), enabled efficient transduction in the presence of pre-existing NAbs at least at a moderate titer. Moreover, exosome encapsulation improved cellular and nuclear uptake of AAV through a non-canonical pathway, which is independent of known receptors. In this study, exo-AAV also enhanced Tregs expansion, and thus induced immune tolerance. Two other studies showed that exosome-encapsulated AAV8 and AAV9 had comparable or improved infectivity in addition to having 3 to 23-fold higher resistance to NAbs (143, 144). Indeed, exo-AAV may have improved tropism to specific tissues. Exo-AAV9 had an improved ability to cross the BBB and target neurons and astrocytes than AAV9 (145). Exo-AAV had improved ability to cross the inner limiting membrane of the retina after intravitreal injection in mice (146). Exo-AAV also efficiently transduced outer hair cells without detectable toxicity (145). It was also shown that exo-AAV was easier and faster to purify as it requires only simple ultracentrifugation of media. One concern about exo-AAV is the reduced purity and the possibility of containing nucleic acids and proteins as shown in other studies using exosome preparations (147). By overexpressing tetraspanin CD9, an exosome marker, the exosome output from AAV-packaging HEK293 cells increased, resulting in a remarkable increase of AAV1 yields (148). Moreover, these vectors had improved transduction compared with standard exo-AAVs. Finally, a similar approach to exo-AAV is epitope masking using small molecules, e.g., albumin. A study in adenovirus showed that when an albumin-binding domain was inserted to capsid proteins of adenovirus, it can protect vectors from NAb recognition while maintaining infectivity and transduction (149). This strategy may be applied to AAV. While all these encapsulation strategies seem to be promising, they also create a challenge for scalable manufacturing and release tests of clinical-grade vectors.

### 3.4 Methods to address cellular immunity

#### 3.4.1 Reduce recognition of unmethylated CpG

Unmethylated CpG elements elicit innate immunity and CTL response through TLR9/MyD88-dependent pathway (23). Recombinant AAV vector has abundant hypomethylated CpG motifs due to the viral or bacterial origin of its sequence compared with human DNA, providing the basis for discriminating AAV and human genome sequences by TLR9. Previous animal studies have shown the immune-activating effects of CpG motifs in AAV (23, 150, 151). For instance, CpG-depleted AAVrh32.33 vectors achieved sustained transgene expression and reduced lymphocyte infiltration in mice (152). A more recent study showed CpG deletion from AAV genome reduced CD8+ T cell response in a murine model of hemophilia after intramuscular delivery (152). Further, a recent study reviewed multiple hemophilia B gene therapy trials and found that sustained transgene expression correlated well with low CpG content in AAV vectors, but not other parameters (reviewed in 153). Since CpG removal is usually performed by synonymous codon substitution, one concern is misfolding of transgene products due to species differences in codon usage (154). Moreover, CpG not only exists in the open reading frame (ORF), but also promoters, enhancers, and ITRs. Removing CpG in those regions may have impacts on functionality of AAV vectors (transcription, packaging). To this end, one study developed methods to generate functional CpG-free ITRs (155), but the yields decreased by 3 fold due to reduced genome replication. Another approach is to increase AAV methylation by improving production technologies. By providing sufficient methyl transferase when preparing input DNA vectors (e.g., plasmids, baculovirus) and packaging AAV in host cells (e.g., HEK293, Sf9), CpG methylation of AAV genome could be achieved.

Alternatively, since CpG elements are recognized by TLR9 (23), inhibition of TLR9 recognition can be also used to reduce CpG-activated cellular immunity. TTAGGG repeat commonly found in mammalian telomeres has been shown to be able to inhibit TLR9 signaling and downstream immune activation (156, 157). Incorporating TLR9 inhibitory sequence (TTAGGG motif) in AAV vectors reduced CTL responses and enhanced transgene expression in mice and pigs (158). However, this strategy only delayed but could not prevent CTL response in NHPs, indicating that immune factors other than TLR9 contributed to intra-ocular inflammation in NHPs. Based on this technology, a startup Ally Therapeutics was launched in 2018. Nevertheless, due to ‘disappointing results in animal trials’, the company was shut down in 2021. Still, the approach represents a novel direction that warrants further investigation.

#### 3.4.2 Induction of Tregs

Tregs are a subset of T cells that suppress immune response (159) and have been implicated in contributing to long-term transgene expression in preclinical studies (35, 160, 161) and clinical trials (162–164). A study in mice demonstrated that coadministration of antigen-specific Tregs and AAV successfully reduced both cellular and humoral immune responses against transgene products, and enabled sustained transgene expression (165). Another study showed that adoptive transfer of *ex vivo* Tregs induced antigen-specific immune suppression and achieved sustained transgene expression after AAV delivery in a murine model of hemophilia B (166). More recently, AAV capsid-specific chimeric antigen receptor Tregs (AAV CAR Tregs) were generated and shown to be able to reduce inflammation, mediate sustained transgene expression, and suppress immune response against both capsids and transgene products after injection into mice (167). In addition to this *ex vivo* Tregs approach, *in vivo* induction of Tregs can be applied to suppress AAV immunity through administration of rapamycin (discussed in section 3.1) or liver gene transfer. It has been shown in multiple preclinical (160, 168–171) and clinical studies (34) that liver-targeted AAV delivery induced immune tolerance of transgene products. In summary, induction of Tregs represent a novel approach to provide lasting and targeted immune suppression, which may be preferred over broad-spectrum and transient immune suppression offered by corticosteroids.

### 3.5 Other methods

#### 3.5.1 Nucleotide sequence to reduce antigen presentation

One viable approach is to limit transgene expression from APCs to reduce antigen presentation and immune responses against AAV gene therapy. This can be done by using tissue-specific promoters or miRNA sequences. Notably, it was recently shown that tissue-specific promoter could not reduce immune responses in a canine study using AAV delivery of the CRISPR system (172). As to miRNA, it has been used to detarget transgene expression from APCs (173, 174). In one study, AAV containing miRNA-142 abrogated CTL and humoral response and enabled sustained transgene expression in mice (173). However, this effect seems to be strain-specific: miRNA-142 attenuated CTL response in WT C57BL/6 mice, but not muscular dystrophy mice (174). Another approach to limit antigen cross presentation is through using a small peptide named ICP47 (175). ICP47, derived from herpes simplex virus, could inhibit MHC class I pathway and antigen cross presentation. Expressing ICP47 from the AAV genome has been shown to reduce CTL responses in mice (176).

#### 3.5.2 Selection of capsid variants or ROAs

Prevalence of AAV2 NAbs in humans was shown to be higher than other capsid variants (28), but cross-reactivity between different capsid variants was relatively low (177). Therefore, using alternative capsid variant with low immunogenicity was shown to be a viable option for achieving efficient transduction in animal studies (178–184). For instance, the first administration of AAV8 in neonatal mice did not prevent a second administration of AAV9 in adults (181). Admittedly, neonatal injection was known to induce immune tolerance (185, 186), making it easier for a second administration. Another study showed that a second administration of AAV6 was not affected by prior administration of AAV2 in adult mice, and vice versa (179). Similarly, increased transgene expression in skeletal muscle was observed after AAV1 administration in AAV2- or AAV5-pretreated mice (180). In addition, AAVrh10-mediated transgene expression was not affected by prior AAV2 or AAV5 administration in mice (182). Capsid variant switch also enabled repeated administration of AAV vectors in rats (183). More importantly, this strategy was shown to be successful in large animal models. In a canine model of hemophilia B, a second administration of AAV8 in AAV2-pretreated dog increased FIX level from <1% to 16% of normal controls and lasted for 2 years (184). Also, no significant liver toxicity and antibody formation was observed in this study. Another approach is to directly use a serotype with low immunogenicity. Since AAV5 is the most structurally divergent of wildtype serotypes, it was hypothesized that AAV5 might be less likely to elicit NAbs (187). One study showed that AAV5 delivery mediated therapeutic level transgene expression in the presence of pre-existing NAbs in NHPs and humans (188). Interestingly, another study found that pre-existing antibody led to significantly reduced transgene expression in NHPs (189).

In addition to capsids, careful selection of route of administration (ROA) can also help overcome AAV immunity. While systemic administration leads to more physical contact between AAV and pre-existing antibodies, local delivery to retina or CNS may be less likely to cause immune responses (190, 191). Notably, one study demonstrated that transgene expression after intrastriatal AAV delivery in rats was inhibited by high circulating NAb titer (1:1,208) (192). Similarly, a mouse study showed that high titer (>1:6,400) circulating NAbs inhibited brain transduction after CNS-directed delivery (190). This study also found that the antibodies in the brain was 0.6% of those in serum, suggesting that a small amount of circulating antibodies cross the blood-brain barrier (BBB). One ramification is that the BBB is compromised in certain neurological diseases (MPS IIIB, Parkinson’s disease) (193, 194), making the inhibitory effects of NAbs more likely. Therefore, this approach should still be considered as long as serum high titer is not extremely high and the BBB is not compromised.

## 4 Discussion

The potential of AAV-mediated gene therapy is significantly limited by immunogenicity. First of all, pre-existing NAbs exclude many patients (> 20% depending on serotypes) from being eligible for the treatment (177). It has also been shown that anti-AAV NAbs could persist for 15 years (195). When the field moves from rare disease, most of which are pediatric diseases, to more common diseases (e.g., Parkinson’s disease), most of which affect adults and even aged population, it becomes a bigger problem because people have a higher chance of being exposed to AAV as they age. Second, immune responses disallow repeated administration of AAV. Although AAV gene therapy is expected to provide long-term efficacy, there may be a need for repeated administration, especially for pediatric patients whose cells continue to expand. Third, AAV immunity could cause severe toxicity, possibly resulting in patient deaths and clinical holds (10, 26, 27). Over the past few years, more treatment emergent serious adverse events (TESAEs) have been reported as more patients receive AAV delivery (6), generating serious concerns about AAV immunogenicity and toxicity.

As mentioned above, there have been extensive efforts to address different aspects of AAV immunity. Many of these approaches have been applied in clinical studies and achieved their goals, at least, to an extent. One strategy, capsid engineering, is particularly promising as it has the potential to develop next generation vectors that have multi-dimensional improvements over currently available vectors. Current AAV vectors are still limited by the low delivery efficiency to specific tissues, resulting in the need of high dose or invasive administration. Improvement in tropism and transduction efficiency alone will enable the usage of lower dose and thus significantly reduce toxicity from AAV immunity. Moreover, next generation vectors with immune-evading features can help address the issue as well.

In this review, the toolkit to overcome AAV immunity are categorized mainly based on methodology, instead of on targets (capsids, genome, or transgene products). Although less discussed in previous sections, immune responses against transgene products should not be underestimated. CTL responses against transgene products were initially observed in some patients of a DMD trial through intramuscular AAV delivery (196). More recently, CTL responses against transgene products and reduced transgene expression were observed in one alpha-1 antitrypsin patient receiving intramuscular AAV delivery (197). Similar observations occurred in MPS IIIB patients receiving intracranial AAV delivery (198). It is worthwhile to mention that immunity against transgene products may vary on genetic backgrounds (34, 199). For instance, AAV gene therapy in individuals who have residual protein expression may be less likely to cause anti-transgene immunity than in those with no residual endogenous expression. This may be further complicated by the fact that some patients of protein deficiencies may have received protein replacement therapies prior to AAV delivery. In addition, tissue inflammation due to the underlying disease can also increase the risk of transgene immunogenicity (200).

A particular challenge for understanding mechanisms of and developing methods to overcome AAV immunity is the species difference between animal models and patients, especially T cell responses. For instance, unlike in mice, there was expansion of pre-existing capsid-specific CD8^+^ T cells in humans (31). Similarly, preclinical studies in mice (92), dogs (201), and NHPs (202) could not recapitulate capsid-specific T cell responses in humans. In addition, many animal models were generated through completely disrupting the function of a certain gene, which may not recapitulate human conditions as some patients still have residual protein expression. This fact adds further complication when using preclinical studies in animals to predict AAV immunity in clinical studies. Therefore, there is a critical need to develop more suitable animal models to predict AAV immunity in humans. Other areas that this field should consider to focus on include: 1) mechanistic studies of toxicity events; 2) identification of reliable preclinical models to monitor and predict AAV immunity and toxicity; 3) standardization of methods that measure AAV immunity, e.g., NAb assays, ELISPOT, complement panel; and 4) long-term follow-up of patients who have received AAV delivery and proper archiving and sharing of these data.

## Author contributions

XL, XW, JL, and LO attended discussion for the project design. XL and LO drafted the manuscript. XW and JL revised the manuscript. All authors contributed to the article and approved the submitted version.

## Funding

This work was supported by National Natural Science Foundation of China (81972204), Natural Science Foundation of Guangdong Province (2019A1515011097), Innovation Program of Shenzhen (Grant No. JCYJ20180508165208399), the grant from the State Key Lab of Respiratory Disease, Guangzhou Medical University (SKLRD-Z-202002), and the 111 Project (D18010) from the Ministry of Education of China. The open research funds from the Sixth Affiliated Hospital of Guangzhou Medical University, Qingyuan People's Hospital.

## Conflict of interest

Author XW was employed by Guangzhou Dezheng Biotechnology Co., Ltd. Author LO was employed by Genemagic Bio.

The remaining authors declare that the research was conducted in the absence of any commercial or financial relationships that could be construed as a potential conflict of interest.

## Publisher’s note

All claims expressed in this article are solely those of the authors and do not necessarily represent those of their affiliated organizations, or those of the publisher, the editors and the reviewers. Any product that may be evaluated in this article, or claim that may be made by its manufacturer, is not guaranteed or endorsed by the publisher.
